# Janus Kinase Inhibitors in the Treatment of Type I Interferonopathies: A Case Series From a Single Center in China

**DOI:** 10.3389/fimmu.2022.825367

**Published:** 2022-03-28

**Authors:** Wendao Li, Wei Wang, Wei Wang, Linqing Zhong, Lijuan Gou, Changyan Wang, Jingran Ma, Meiying Quan, Shan Jian, Xiaoyan Tang, Yu Zhang, Lin Wang, Mingsheng Ma, Hongmei Song

**Affiliations:** Department of Pediatrics, Peking Union Medical College Hospital, Chinese Academy of Medical Sciences, Peking Union Medical College, Beijing, China

**Keywords:** type I interferonopathies, autoinflammatory disorders, Janus kinase inhibitors, ruxolitinib, tofacitinib

## Abstract

**Objective:**

This study aimed to assess the efficacy and safety of 2 Janus kinase (JAK) inhibitors (jakinibs) tofacitinib and ruxolitinib in the treatment of type I interferonopathies patients including STING-associated vasculopathy with onset in infancy (SAVI), Aicardi-Goutières syndrome (AGS), and spondyloenchondrodysplasia with immune dysregulation (SPENCD).

**Methods:**

A total of 6 patients were considered in this study: 2 patients with SAVI, 1 patient with AGS1, 1 patient with AGS7, and 2 patients with SPENCD. Clinical manifestations, laboratory investigations, radiology examinations, treatment, and outcomes were collected between November 2017 and November 2021 in Peking Union Medical College Hospital. The disease score for patients with SAVI and AGS scale for patients with AGS were documented. The expression of 6 interferon-stimulated genes (ISGs) was assessed by real-time PCR.

**Results:**

Three patients (1 patient with SAVI, 2 patients with AGS) were treated with ruxolitinib and 3 patients (1 patient with SAVI, 2 patients with SPENCD) were treated with tofacitinib. The mean duration of the treatment was 2.5 years (1.25–4 years). Upon treatment, cutaneous lesions and febrile attacks subsided in all affected patients. Two patients discontinued the corticoid treatment. Two patients with SAVI showed an improvement in the disease scores (*p* < 0.05). The erythrocyte sedimentation rate normalized in 2 patients with AGS. The interferon score (IS) was remarkably decreased in 2 patients with SPENCD (*p* < 0.01). Catch-ups with growth and weight gain were observed in 3 and 2 patients, respectively. Lung lesions improved in 1 patient with SAVI and remained stable in 3 patients. Lymphopenia was found in 3 patients during the treatment without severe infections.

**Conclusion:**

The JAK inhibitors baricitinib and tofacitinib are promising therapeutic agents for patients with SAVI, AGS, and SPENCD, especially for the improvement of cutaneous lesions and febrile attacks. However, further cohort studies are needed to assess the efficacy and safety.

## Introduction

Type I interferon (IFN)-mediated monogenic autoinflammatory (IFNopathies) disorders are recently identified as a subgroup of inborn errors of immunity, which include a genetically and phenotypically heterogeneous group of autoinflammatory and autoimmune disorders with high morbidity and mortality. They predominantly affect young child such as Aicardi–Goutières syndrome (AGS), STING-associated vasculopathy with onset in infancy (SAVI), and spondyloenchondrodysplasia with immune dysregulation (SPENCD) (1, 2). According to the last classification from the International Union of Immunological Societies Expert Committee, 15 different gene mutations correspond to 15 distinct disorders and many IFN-related diseases have yet to be discovered. IFNopathies are different from the inflammatory diseases mediated by interleukin (IL)-1 and tumor necrosis factor (TNF)-α, since they are characterized by the overproduction of type I IFN in the blood and cerebral spinal fluid, leading to the excessive activation of Janus kinase (JAK)/signal transducer and activator of transcription (STAT) pathway (3). Clinical manifestations are also distinct from those of the canonical autoinflammatory disorders. Intracranial calcification especially in basal ganglia, interstitial lung disease, and early onset of the skin vasculopathy with chilblains and livedo reticularis are the most represented common features (4). The treatment is also distinctive and challenging. Furthermore, so far there is no consensus on the treatment of this type of disease. More and more studies found that IFNopathies poorly respond to conventional immunosuppressive treatments (5, 6). Thus, highly efficacious drugs are urgently needed.

JAK inhibitors (jakinibs) block the activation of the IFN pathway by inhibiting JAKs, resulting in a very promising therapeutic strategy for adults suffering from autoimmune, inflammatory, and hematological pathologies such as rheumatoid arthritis, psoriasis arthritis, and ulcerative colitis (7). In light of the pathogenesis of IFNopathies, the inhibition of the JAK activity shed light on a novel treatment target in these disorders. Indeed, several studies mostly in patients with SAVI and chronic atypical neutrophilic dermatitis with lipodystrophy (CANDLE) revealed that jakinibs improve the symptoms, control the disease activity, and suppress the IFN signature in most IFNopathy genotypes (8). However, the long-term outcome remains to be evaluated. Therefore, in this work, the outcomes of the treatment with jakinibs of 6 IFNopathy patients from a single center in China including 2 patients with SAVI, 2 patients with SPENCD, 1 patient with AGS1, and 1patient with AGS7 were reported.

## Methods

### Patient Cohort and Study Approval

A retrospective analysis was performed on 6 patients who suffered from genetically confirmed IFNopathies between November 2017 and November 2021 in the Department of Pediatrics in Peking Union Medical College Hospital. All diagnoses were made by next-generation sequencing (NGS) and validated by Sanger sequencing. Age, gender, clinical manifestation, disease course, treatment, side effects, and laboratory data were collected from the electronic database. Some routine tests were performed at each follow-up visit including blood count, liver and kidney function tests, urine routine analysis, C-reactive protein (CRP), erythrocyte sedimentation rate (ESR), immunoglobulin level, complement, lymphocyte subset panels, and autoantibody tests. Chest high-resolution computed tomography, brain magnetic resonance imaging (MRI), cranial computerized tomography, plain X-ray, pulmonary function tests (PFTs), and 6-min walking test were also carried out every 6 or 12 months, depending on each patient, to evaluate the disease conditions. The study was approved by the Peking Union Medical College Hospital ethics committee and performed in accordance with the Declaration of Helsinki. Written informed parental consent was obtained for the use of jakinibs in the presence of a lawyer, and the consent of the parents was obtained for conducting the experiments.

### Disease Activity Score

Disease activity rating scale of SAVI patients was evaluated according to the method described by Crow et al. (9), was used to evaluate the disease activity, and was determined at baseline as well as at each visit. The disease activity score of AGS patients was assessed by the AGS scale according to the method described by Adang (10) at each visit or based on the parental recall.

### IFN Signature Assessment

The peripheral blood was collected into EDTA tubes. Total RNA was then extracted from the whole blood by RNA iso Plus (TaKaRa, Japan) following the manufacturer’s instructions. RNA concentration was assessed by a spectrophotometer (Thermo Nanodrop 2000, USA). cDNA was derived from 200 ng total RNA and then quantitative reverse transcription polymerase chain reaction (qRT-PCR) was performed in duplicate in 96-well plate using a SYBR Green Master Mix kit (Applied Biosystems, USA) and ABI7500 PCR system (Applied Biosystems, San Francisco, CA, USA). We studied 6 ISGs in the blood (IFIT1, IFI27, IFI44L, ISG15, SIGLEC1, RSAD2) as previously described (11). The relative expression was calculated by the 2^−ΔΔCt^ method and normalized to the geometric mean of the expression of 2 housekeeping genes: β-actin and OAZ. The sequence of the primers is listed in **Supplementary Table S1**. For each of the 6 ISGs, individual data were expressed relative to a single calibrator (pool of 28 healthy controls). The median fold change of the 6 ISGs was determined as an interferon score (IS) for each patient. An abnormal IS was defined as greater than +2 standard deviations above the mean of the control group (>2.56). IS was assessed before jakinibs treatment as well as at each follow-up visit.

### Statistical Analysis

Statistical analysis was performed using GraphPad Prism software (version 8.0.1, GraphPad Inc.). Data were expressed as median (minimum-maximum range). Unpaired *t*-test and one-way ANOVA were used to compare two or more groups, respectively. A value of *p* < 0.05 was considered statistically significant.

## Results

A total of 6 patients were included in our study: 2 patients with SAVI, 2 patients with SPENCD, 1 patient with AGS1, and 1 patient with AGS7. Among them, 3 were women. The median age of the disease onset was 1.8 years old (0.3–12.7 years old). The mean interval between the disease onset and the final genetic diagnosis was 5.6 years (0.1-12.3 years) and the mean interval between the disease onset and the start of the treatment with jakinibs was 6.6 years (1.1–13.5 years). The mean duration of the treatment was 2.5 years (1.25–4 years) (**Table 1**).

**Table 1:** Clinical manifestations, treatment of IFNopathies patients.

	Patient 1	Patent 2	Patient 3	Patient 4	Patient 5	Patient 6
Sex	M	M	F	M	F	F
Current age	3 years and 4 months	18 years	16 years and 9 months	5 years and 7 months	14 years and 7 months	17 years and 5 months
Age of onset	3 months	6 months	3 years	6 months	4 years	12 years and 8 months
Age of diagnosis	1 year and 3 months	12 years and 10 months	13 years and 4 months	2 years and 6 months	11 years and 11 months	13 years
Age of jakinibs treatment	1 years and 4 months	14 years	13 years and 4 months	3 years and 1 month	13 years and 4 months	15 years and 7 months
Mutation	TMEM173: p. N154S	TMEM173: p.V155M	TREX1: p. G47S; p.C154Mfs*3	IFIH1: p. A339D	ACP5: p. G215R; p. L247P	ACP5: p. S267Lfs*20; p. G239D
Diagnosis	SAVI	SAVI	AGS1	AGS7	SPENCD	SPENCD
Systemic inflammation	+	+	+	+	−	+
Febrile attacks	+	−	+	+	−	+
Failure to thrive	Wt < −2SD	Wt < −2SD	−	Wt < −2SD, Ht < −2SD, Hc < −2SD	−	Ht < −2SD
Infections	Pneumonia	Pneumonia	Cytomegalovirus, recurrent respiratory tract infections	*Staphylococcus aureus*, *Candida albicans*	−	−
Cutaneous manifestations	Chilblain-like lesions, ulcers, erythematous rashes	Chilblain-like lesions, angiotelectasis	Chilblain-like lesions, erythematous rashes	Chilblain-like lesions, erythematous rashes, ulcers, livedo reticularis	−	−
Neurological manifestations	−	−	Basal ganglia calcification, brain infarction, cognitive impairment, extrapyramidal symptoms	Basal ganglia calcification, leukodystrophy	Encephalalgia, intracranial calcification, leukodystrophy, middle cerebral artery occlusion, extrapyramidal symptoms	Intracranial calcification, leukodystrophy, extrapyramidal symptoms
Respiratory manifestations	Cough, tachypnea, dyspnea, hypoxemia, digital clubbing	Cough, tachypnea, dyspnea, hypoxemia, wheezing, crackles, cyanosis, digital clubbing, pulmonary arterial hypertension	−	−	−	−
Interstitial lung disease	+	+	−	+	−	+
Skeletal manifestations	−	−	−	−	Platyspondyly, metaphyseal dysplasia	Short stature, platyspondyly, metaphyseal dysplasia
Other features	−	Right ventricular enlargement	Glaucoma	Hypothyroidism	IgA nephropathy	Liver calcifications, hypothyroidism, AIH
Treatment before jakinibs	Prednisone	Ambrisentan, tadalafil	−	Methylprednisolone, IVIG	−	Prednisone, hydroxychloroquine, levothyroxine sodium, IVIG, MMF
Jakinibs	Tofacitinib (0.50 mg/kg/day), ruxolitinib (0.50 mg/kg/day)	Tofacitinib (0.38 mg/kg/day)	Ruxolitinib (0.25 mg/kg/day)	Ruxolitinib (0.71 mg/kg/day)	Tofacitinib (0.24 mg/kg/day)	Tofacitinib (0.22 mg/kg/day)
Concomitant treatment	Prednisone, thalidomide	Prednisone, ambrisentan, tadalafil, theophylline, sulfamethoxazole	Prednisone, thalidomide, aspirin	Prednisone, thalidomide, levothyroxine sodium	−	Prednisone, hydroxychloroquine, levothyroxine sodium

M, male; F, female; SAVI, STING-associated vasculopathy with onset in infancy; AGS, Aicardi–Goutières syndrome; SPENCD, spondyloenchondrodysplasia with immune dysregulation; Wt, weight; Ht, height; SD, standard deviation; IVIG, intravenous immunoglobulin; +, presence; −, absence.

### Clinical Manifestations

**Figure 1:**
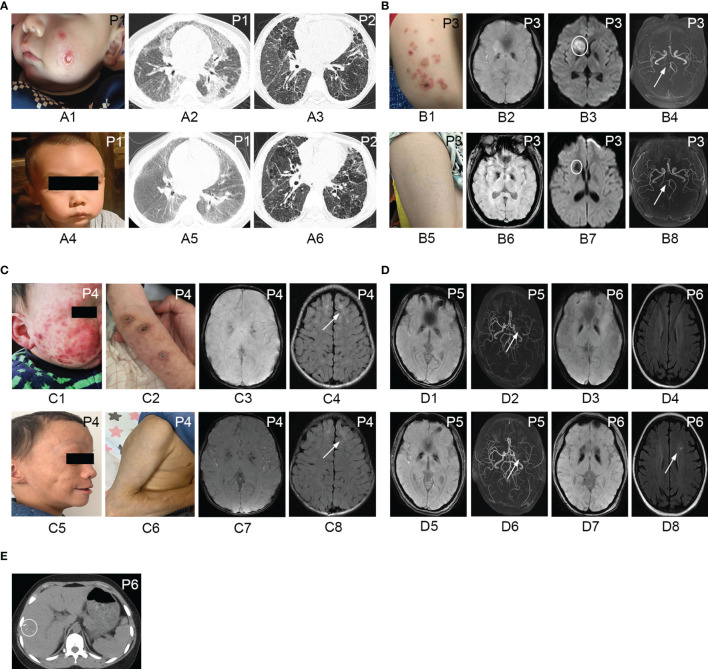
Cutaneous manifestations, chest CT, and brain MRI of patients with IFNopathies before and after the treatment with jakinibs. Chilblain-like lesions and ulcerations on the cheek of patient 1 **(A1)** disappeared after the treatment with ruxolitinib **(A4)**. Chest CT scan of patient 1 demonstrating diffuse cords, patchy consolidation, and ground-glass opacities **(A2)** before the treatment, which improved after the treatment **(A5)**. Chest CT scan showing no significant improvement of reticular opacities, ground-glass opacities, and cysts in patient 2 before **(A3)** and after **(A6)** the treatment with tofacitinib. Erythematous rashes on the left leg of patient 3 **(B1)** resolved after the treatment with ruxolitinib **(B5)**. Susceptibility weighted imaging showing symmetric hypointense signal in the basal ganglia region in patient 3 **(B2)**, patient 4 **(C3)**, patient 5 **(D1)**, and patient 6 **(D3)** corresponding to calcification areas in brain CT scan and no improvement observed after the treatment **(B6, C7, D5, D7)**. Diffusion weighted imaging showing hyperintensity in the right head of the caudate nucleus and right anterior limb of the internal capsule **(B3)** in patient 3. Repeated MRI showing hypointense signal in diffusion weighted imaging **(B7)** indicating encephalomalacia after the treatment. Brain magnetic resonance angiography indicating stenosis and occlusion of the posterior cerebral artery **(B4)** in patient 3 and recanalization after the treatment **(B8)**. Erythematous rashes, chilblain-like lesions, ulcerations on the cheeks, and the arm of patient 4 **(C1, C2)** disappeared after the treatment with ruxolitinib, left with subcutaneous lipoatrophy **(C5, C6)**. T2 FLAIR MRI showing hyperintensity in the bilateral frontal lobe in patient 4 **(C4)** and no improvement after the treatment with ruxolitinib **(C8)**. Brain magnetic resonance angiography reveling occlusion of the left middle cerebral artery with the formation of peripheral collateral circulation with no changes before **(D2)** and after the treatment **(D6)** in patient 5. T2-weighted MRI of patient 6 demonstrating hyperintensity in the left corona radiata **(D8)** the after treatment with tofacitinib which was not observed before the treatment **(D4)**. Abdominal CT scan showing multiple calcifications in the liver in patient 6 **(E)**. P, patient.

The most common features were febrile attacks and chilblain-like lesions which could be seen in 4 out of 6 patients (66.7%), respectively (**Figures 1A1, B1, C1**). Patient 2 also showed angiotelectasis in the back, and livedo reticularis was found in patient 4 who suffered from repeated ulcers and erythematous lesions in the cheeks and the arms as well (12) (**Figures 1C1, C2**). Failure to thrive is another universal characteristic. Patients 1 and 2 were below −2 standard deviation (SD) for weight; patient 6 was below the −2SD for height; and patient 4 was below the −2SD for weight, height, and head circumference. Patients 4 and 6 manifested with motor development delay, and patient 4 also showed language development delay. Other neurological features included congenital impairment in patient 3 as evidenced by narcolepsy, reduced memory, calculation, concentration, and academic performance, and limp in patients 3, 5, and 6. Four patients experienced infection susceptibility. Recurrent pneumonia occurred in patients 1 and 2. Patient 3 experienced recurrent respiratory tract infections at the early age of childhood, and *Staphylococcus aureus* and *Candida albicans* infection occurred in patient 4 who manifested severe skin vasculopathy. Other features included right ventricular enlargement (patient 2), glaucoma (patient 3), recurrent encephalalgia, and IgA nephropathy (patient 5). Patient 6 was initially diagnosed with autoimmune hepatitis according to the liver dysfunction, positive liver-kidney microsomal antibody, and liver puncture biopsy revealing moderate-to-severe interface hepatitis (**Table 1**).

Both SAVI patients were subjected to respiratory symptoms from the disease onset including cough, tachypnea, dyspnea, hypoxemia, and digital clubbing (**Table 1**). Patient 2 also presented with wheezing, crackles, cyanosis, and pulmonary hypertension (PH) due to the severe lung disease; thus, a lung biopsy was performed before the genetic diagnosis, revealing dilated alveolar cavity with a large number of foamy histiocytes, type II epithelial cell hyperplasia, widened alveolar septa, and fibrous tissue hyperplasia. In addition, right heart catheterization indicated precapillary PH. PFTs of patient 2 was also performed. His forced expiratory volume in 1 s was 31.6% predicted, and his diffusing capacity of the lungs for carbon monoxide was 24.1% predicted, which revealed severe restrictive lung function defect with reduced diffusion capacity.

### Laboratory Investigations

Laboratory parameters showed elevated CRP in 2 patients and elevated ESR in 4 patients. Decreased white blood cell and neutrophil were present in 2 patients. Anemia was found in 1 patient, and lymphopenia was detected in 2 patients. The urine routine analysis revealed proteinuria and hematuria in 1 patient. Hypergammaglobulinemia was found in 4 patients. Complement 3 decreased in 2 patients. Alanine aminotransferase and aspartate transaminase were increased in 4 patients. Positive expression of antinuclear antibodies, antidouble-stranded DNA, antineutrophil cytoplasmic antibodies, rheumatoid factors, anticyclic citrullinated peptide, antihistone antibodies, and antiribonucleoprotein was found in 5 patients. Thyroid function tests revealed an increased thyroid-stimulating hormone in 3 patients (**Table 2**).

**Table 2:** Laboratory parameter changes before and after jakinibs treatment.

	Patient 1		Patient 2		Patient 3		Patient 4		Patient 5		Patient 6	
	M0	Mmax	M0	Mmax	M0	Mmax	M0	Mmax	M0	Mmax	M0	Mmax
Blood count	N	WBC, NEU#, ALC↓	N	ALC↓	N	N	WBC, NEU#↓	N	N	NEU#, ALC↓	WBC, NEU#, ALC, HgB↓	WBC, NEU#, ALC, HgB↓
CRP (mg/L)	13	10	12	14	2	5	4	1	1	1	1	2
ESR (mm/h)	28	21	78	84	25	6	44	15	6	8	19	32
Urine routine	N	N	N	N	Proteinuria, hematuria	N	N	N	N	N	N	Proteinuria
Liver function	N	N	N	N	ALT, AST↑	N	ALT, AST↑	N	N	N	ALT, AST↑	N
IgG (g/L)	13.08↑	18.07↑	22.16↑	29.07↑	17.46↑	9.87	24.27↑	17.49↑	14.19	14.94	8.72	13.66
IgA (g/L)	0.9	3.63↑	4.73↑	6.46↑	2.68↑	1.72	4.73↑	4.51↑	4.48↑	5.02↑	4.17↑	5.79↑
IgM (g/L)	1.19	1.07	0.72	0.71	0.75	0.34↓	0.56	0.73	0.82	0.77	0.16↓	0.24↓
Complement	C3↓	N	N	N	C3↓	N	N	N	N	N	N	N
Lymphocytes (/µl)	3,920	980↓	2,812	1,320↓	NA	NA	2,030↓	NA	3,001	1,420↓	263↓	341↓
CD3+ lymphocytes (/µl)	3,066	662↓	2,078	810↓	NA	NA	1,624↓	NA	1,651	723↓	198↓	287↓
CD3+CD4+lymphocytes (/µl)	976	160↓	793	269↓	NA	NA	646↓	NA	684	293↓	90↓	129↓
CD3+CD8+lymphocytes (/µl)	2,011	476↓	1,234	536	NA	NA	924	NA	786	352↓	98↓	144↓
B cells (/µl)	710	268↓	574↑	261	NA	NA	35↓	NA	1,077↑	656	19↓	39↓
NK cells (/µl)	63↓	67↓	152↓	285	NA	NA	309	NA	237	24↓	43↓	12↓
Autoantibodies	ANA, dsDNA, ANCA, AHA, CCP, RF	ANA	RF	N	ANA, dsDNA	ANA	N	N	RNP, RF	AHA, RF	ANA	ANA, Coombs
Thyroid function	NA	NA	N	N	N	N	TSH↑	N	TSH↑	N	TSH↑	TSH↑
Interferon score	21.42	32.3	NA	17.41	NA	4.95	23.11	21.79	135.54	19.67	36.13	19.46

WBC, white blood count; NEU#, neutrophil; HgB, hemoglobin; ALC, absolute lymphocyte count; CRP, C-reactive protein; ESR, erythrocyte sedimentation rate; ALT, alanine transaminase; AST, aspartate transaminase; Ig, immunoglobulin; ANA, antinuclear antibodies; dsDNA, antidouble stranded DNA; AHA, antihistone antibodies; CCP, anticyclic citrullinated peptide; RF, rheumatoid factors; RNP, antiribonucleoprotein; TSH, thyroid-stimulating hormone; ↑, higher than the normal range; ↓, lower than the normal range; M0, the time before jakinibs treatment; Mmax, time at the last follow-up visit; N, normal; NA, not available.

### Imaging Examinations

Intracranial calcification was the predominant feature in IFNopathy patients. The symmetrical calcification of the basal ganglia was found in 4 patients, but not in the 2 SAVI patients (**Figures 1B2, C3, D1, D3** and **Supplementary Figures S1A–D**). In addition to intracranial calcification, patient 6 showed multiple liver calcification (**Figure 1E**). Patient 3 also presented with cerebral infarction in the right head of the caudate nucleus, right anterior limb of the internal capsule, and stenosis of the posterior cerebral artery (**Figures 1B3, B4**). Patient 5 presented middle cerebral artery occlusion as well (**Figure 1D2**). Leukodystrophy could be seen in 3 patients (**Figure 1C4**). Plain X-ray revealed the presence of platyspondyly and metaphyseal dysplasia in both patients 5 and 6 (**Supplementary Figures S1E, F**). Chest CT scan in patient 1 revealed diffuse cords, patchy consolidation, and ground-glass opacities (**Figure 1A2**). Multiple cords, reticular opacities, ground-glass opacities, and cysts were found predominantly in the lower lobes of patient 2 (**Figure 1A3**). Mild bilateral interstitial lung disease also occurred in patients 4 and 6 (**Supplementary Figures S2A, B**).

### ISG and IS Analysis

ISG expression was evaluated in 5 patients prior to jakinib treatment, and all of them showed a dramatically increased IS with a median of 29.62 (21.42–135.54) compared with healthy controls (*p* < 0.0001).

## Treatment and Outcomes

### Treatment

**Figure 2:**
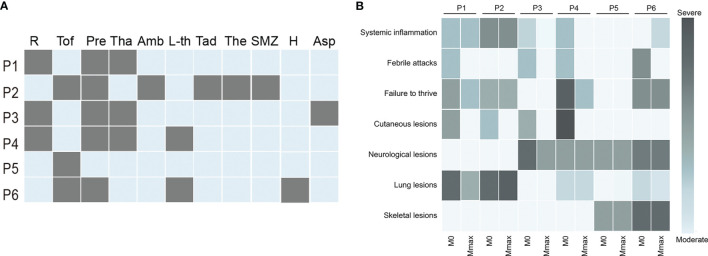
Treatment and prognosis of patients with IFNopathies. **(A)** The treatment of patients with IFNopathies. Each row represents a patient, and each column represents a drug. A grey box indicates the presence of this treatment. **(B)** The change of clinical manifestations before and after jakinib treatment. Each row represents a manifestation, and each column represents a patient. The shade of color indicates the severity of the symptoms. The light blue box represents the absence of this symptom. P, patient; R, ruxolitinib; Tof, tofacitinib; Pre, prednisone; Tha, thalidomide; Amb, ambrisentan; L-th, levothyroxine sodium; Tad, tadalafil; The, theophylline; SMZ, sulfamethoxazole; H, hydroxychloroquine; Asp, aspirin; M0, the time before jakinibs treatment; Mmax, the time at the last follow-up visit.

Patients 1, 2, 5, and 6 received tofacitinib with a median dosage of 0.26 (0.12–0.3) mg/kg/day at start, and the dosage was gradually escalated according to the treatment response. Patient 5, who predominantly suffered from headache and extrapyramidal symptoms without detectable systemic inflammation all the time, was also treated with jakinibs considering the neurological manifestations and the dramatically increased IS. Patients 3 and 4 were subjected to ruxolitinib treatment with a dosage of 5 mg twice daily and 2.5 mg twice daily, respectively. The choice of jakinibs depended on the availability of drugs and the affordability of the patients. Tofacitinib was replaced with ruxolitinib in patient 1 after 15 months due to the unsatisfied control of CRP and ESR. All patients were also subjected to a treatment with a combination of corticosteroids, with the exception of patient 5 who never demonstrated any systemic inflammation. Although the cutaneous lesions and febrile attacks were considerably controlled, ESR was a consequence of the systemic inflammation that remained increased in patients 1, 3, and, and the dosage of jakinibs was already high enough. In addition, considering the side effects in the long-term use of corticosteroids, a concomitant treatment with thalidomide was prescribed for patients 1, 3, and 4 for the suppression of the systemic inflammation. Patient 2 also received anti-PH treatment. Other treatments included aspirin (patient 3), levothyroxine sodium (patients 4 and 6), and hydroxychloroquine (patient 6) (**Table 1** and **Figure 2A**).

### Improvement of Clinical Symptoms After Treatment With Jakinibs

**Figure 3:**
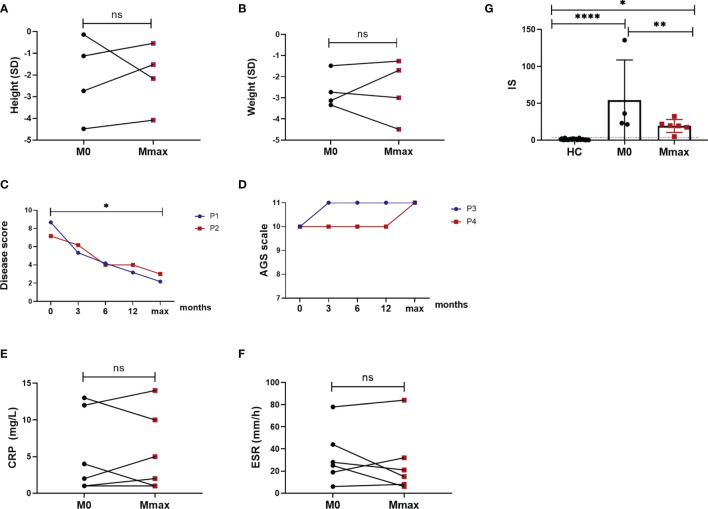
Response to jakinib treatment. Height in standard deviation (SD) **(A)** showing catch-up growth in 3 patients and further failure to thrive in 1 patient (ns). Weight **(B)** in SD showing weight gain in 2 patients and further growth failure in 2 patients (ns). Disease score of 2 patients with SAVI improved after the treatment (*p* < 0.05) **(C)**. AGS scale elevated and stayed stable during the treatment in patient 3 and patient 4 **(D)**. Measures of CRP **(E)** and ESR **(F)** showing no improvement after the treatment (ns). Six gene-based IFN score **(G)** decreased after the treatment (*p* < 0.01). The dotted line indicates the cutoff value (2.56). HC, healthy control; M0, the time before jakinib treatment; Mmax, the time at the last follow-up visit; P, patient; ^*^ *p* < 0.05; ^**^ *p* < 0.01; ^****^ *p* < 0.0001; ns, nonsignificant.

Febrile attacks and cutaneous lesions subsided in all patients after treatment with jakinibs (**Figures 1A4, B5, C5, C6, 2B**). Of note, the most prompt improvement of rashes was observed within 1 month after the start of jakinibs. The rashes completely disappeared in patients 2 and 3 and appeared only in winter in patients 1 and 4 with less severity and duration. However, subcutaneous lipoatrophy remained after remission of rashes in patient 4. Patients 2 and 3 were completely weaned from the treatment of corticosteroid. The median dosage of prednisone dropped to 0.20 (0.11–0.36) mg/kg/day in the remaining 3 patients. Four patients who had growth failure at baseline also got some improvement, although not statistically significant. Catch-up with growth was observed in patient 2 (−1.12 SD to −0.54 SD), patient 4 (−2.73 SD to −1.54 SD), and patient 6 (−4.48 SD to −4.08 SD). Weight gain was observed in patient 1 (−3.13 SD to −1.7 SD) and patient 6 (−1.48 SD to −1.26 SD) (**Figures 3A, B**). The normalization of head circumference (−3.15 SD to −1 SD) was also observed in patient 4. However, patient 1 developed further insufficiency for height, patients 2 and 4 developed further insufficiency for weight. The limp of 3 patients remained stable, and no extra neurological symptoms developed during the treatment.

The disease score of 2 patients with SAVI was also significantly reduced (*p* < 0.05) (**Figure 3C**). Respiratory symptoms alleviated significantly with a considerable amelioration of cough, dyspnea, cyanosis, and tachypnea, and the daily activities became largely limitless for both patients. The digital clubbing of patient 1 also achieved significant remission, and no digital loss occurred. Repeated right heart catheterization in patient 2 resulted in a reduction of PH from 57 mmHg at baseline to 32 mmHg at the last visit. His forced expiratory volume in 1 s on PFTs improved from 31.6% predicted at baseline to 35% predicted after the treatment and the diffusing capacity of the lungs for carbon monoxide remained stable to 24% predicted. The 6-min walking test remained stable with a distance of 510 m. In addition to the amelioration of fevers and rashes, the AGS scale of patients 3 and 4 experienced an elevation to full score and kept stable in the following visit (**Figure 3D**). Patient 3, who showed loss of ambulation and severe cognitive impairment as evidenced by narcolepsy, as well as reduced memory, calculation, and concentration, recovered rapidly and sustainably at 1 week after ruxolitinib treatment. The gross motor and language development of patient 4 also improved. Patient 5 no longer experienced headaches after the treatment with tofacitinib.

### Heterogeneous Improvement of Laboratory Parameters and IS

No significant change in CRP levels was observed in all patients. It fluctuated during the treatment and remained high in 2 patients at the last visit. In patients with normal CRP levels at baseline had no significant upward trend (**Figure 3E**). While the reduction of ESR was not significant after the treatment (**Figure 3F**), patients 3 and 4 finally got a normalization of ESR after the treatment with ruxolitinib. Nevertheless, patient 6 whose ESR level was normal at baseline experienced an increase of ESR at the last visit without any complaints and detected infections. Blood count improved in patient 4 and was reduced in 3 patients. Patient 6 with anemic and lymphopenia at baseline did not experience any increase after the treatment. Proteinuria and hematuria were also quickly under control in patient 3, while patient 6 developed proteinuria during the last visit. Liver function continuously improved with the treatment, reaching a normal range in all patients. C3 levels also returned to normal. Hypergammaglobulinemia did not show any improvement. Autoantibodies were reduced in patients 1, 2, and 3 (**Table 2**). However, a positive Coombs test was found in patient 6. IS also significantly decreased (*p* < 0.01) during the treatment but remained elevated (**Figure 3G**).

### Stable of Imaging Examinations After Jakinib Treatment

The repeated chest CT scan of 4 patients revealed a remarkable improvement in patient 1 (**Figure 1A5**), and no noticeable change was observed in patient 2 (**Figure 1A6**), patient 4, and patient 6 (**Supplementary Figures S2D, E**). Bilateral basal ganglia calcifications found in 4 patients at baseline were not reduced in any of the repeated brain MRI (**Figures 1B6, C7, D5, D7**). Enephalomalacia on MRI was also reduced, and stenosis of the cerebral artery disappeared completely in patient 3 (**Figures 1B7, B8** and **Supplementary Figures S2C, F**). Hyperintensity in the bilateral frontal lobe indicating leukodystrophy in patient 4 remained existent (**Figure 1C8**). The occlusion of the left middle cerebral artery in patient 5 still existed (**Figure 1D6**). However, T2-weighted MRI in patient 6 demonstrated new hyperintensity, which was not observed before (**Figure 1D4**), in the left corona radiata after the treatment with tofacitinib (**Figure 1D8**).

### Side Effects

Overall, tofacitinib and ruxolitinib were well tolerated in all patients. No severe side effects were documented, and no deaths were reported. Only a transient lung infection was reported in patient 1. Patient 3 suffered from cytomegalovirus infection at the beginning of ruxolitinib treatment, but this infection did not aggravate further after the treatment. Although 4 patients developed lymphopenia, which could predispose them to infections, there were no infections detected on regular screening during the treatment.

## Discussion

IFNopathies are a group of rare inherited autoinflammatory diseases characterized by a constitutive overproduction of type I IFN, which is due primarily by defects in the genes related to nuclear sensing, metabolism, and negative regulation of type I IFN pathway. These IFNopathies include the well-known AGS, SAVI, and CANDLE (13). Ten years have passed since the term “IFNopathies” was coined. It is suggested that as many as 38 genetic defects are currently responsible for this group of diseases (14). Timely diagnosis and treatment are critical for patients’ prognosis due to the high morbidity and mortality of these diseases. However, most of these diseases are resistant to conventional immunosuppressive therapy. Jakinibs, including tofacitinib, a blocker of JAK 1/3, and ruxolitinib and baricitinib, blockers of JAK 1/2, have recently shown favorable results in the treatment of these types of diseases (5, 8, 10, 15). Our study further confirmed that jakinibs could be considered a promising therapeutic option for IFNopathies not only for SAVI and CANDLE but also for AGS and SPENCD. Our results demonstrated that the treatment with jakinibs, tofacitinib or ruxolitinib, reduced disease flare-ups and improved symptoms. All patients experienced a significant improvement in cutaneous lesions except patients 5 and 6 who never developed rashes (**Figure 2B**). Three patients were able to permanently free from corticosteroid treatment without disease aggravation. Of note, patient 4 was left with lipoatrophy after the rashes subsided (**Figures 1C5, C6**). Four patients with recurrent episodes of fever completely normalized their body temperature. Intriguingly, patient 2 developed fever again after taking prednisone, and his temperature improved after the withdrawal from it, which was never reported in previous studies. Failure to thrive, another striking feature of IFNopathies, showed heterogeneous improvement in our patients (**Figures 3A, B**).

For SAVI patients, both individuals got amelioration of disease activity. Patient 1, who changed the treatment from tofacitinib to ruxolitinib after 15 months, experienced a dramatic improvement of the lung lesions and rashes after ruxolitinib started, suggesting that tofacitinib, to some extent, is not the best choice for patients with SAVI. Actually, a similar phenomenon was observed in a recent study where tofacitinib failed to halt disease deterioration in 3 patients with SAVI (16). Conversely, ruxolitinib showed considerable results in other studies (6, 9, 17). Although no significant improvement was observed in patient 2 under the concomitant treatment with tofacitinib with ambrisentan and tadalafil, no further exacerbation of the interstitial lung disease was observed, including the stabilization of PFTs, 6-min walking test, and improvement in PH. The variability of disease severity, inadequate plasma drug levels, organ impaired degree prior to jakinib treatment, and the genotype may also contribute to the heterogeneous improvement of this disease (17–19), suggesting that the treatment should be initiated as soon as possible to avoid an irreversible lung damage (20). Intracranial calcification is another universal imaging feature of IFNopathies, found in 4 of our patients. Moreover, patient 6 showed multiple liver calcifications, which were not reported before and might be associated with hepatocellular injury. Although the neurological symptoms improved, the calcifications continuously existed during jakinib treatment. The ability of the drug to penetrate the blood–brain barrier might play a pivotal role. Some studies have found that the concentration of ruxolitinib in the cerebral spinal fluid was only 10%–15% of that in the plasma (21, 22). This inability of the drugs to cross the blood–brain barrier should be taken into consideration when choosing the treatment of patients with neurological symptoms. Of note, the 2 patients with SPENCD in our study demonstrated different responses to the treatment with tofacitinib. Patient 5 achieved a considerable remission after the treatment. However, patient 6 experienced an unsatisfactory disease control as evidenced by increase of ESR, proteinuria, new cerebral lesions, and persistent lymphopenia, although the patients did not have any subjective complaint. Only 1 patient with SPENCD has been reported to date who received baricitinib treatment and achieved symptom control (23). Therefore, more studies are needed to assess the efficacy of jakinibs in patients with SPENCD.

Despite the significant improvement of disease signs and symptoms under the treatment of jakinibs, the systemic inflammatory markers (ESR and CRP) hardly returned to normal, in accordance with previous studies, indicating that many other cytokines besides type I IFN may also play a role in the development of the disease (5). This also suggests that a combined treatment is necessary for these patients. In our study, although no significant decrease in CRP and ESR was observed (**Figures 3E, F**), patients 3 and 4 finally reached the normalization of ESR. In addition to ruxolitinib and corticosteroid, both patients received a combined treatment with thalidomide, then CRP and ESR levels continuously decreased after. A decrease in CRP and ESR was also observed in patient 1 with the addition of thalidomide, albeit they remained above the normal levels. Our study suggests that thalidomide could be a recommended concomitant drug when inflammatory markers are consistently high. Recent studies found that thalidomide can reduce the level of cytokines and antiangiogenic factors and has a strong immunomodulatory effect. It is now widely used in rheumatoid arthritis, inflammatory bowel disease, and cancers, showing encouraging results (24). However, the number of patients in our study was too small and a larger cohort is needed to validate our results. On the other hand, the incomplete inhibition of the JAK-STAT pathway might also contribute to a suboptimal control of CRP and ESR levels (9). IS in our study decreased in most patients during the treatment with jakinibs, in line with previous studies, with a magnitude of the decrease varying from patient to patient and being more pronounced in patients with SPENCD. Although IS decreased, the absolute levels remained elevated (**Figure 3G**). On the contrary, patient 1 whose symptoms were well controlled, experienced an increase of IS in the last test. Several factors might lead to unparalleled symptom improvement and IS. The variable and incomplete reduction of IS could be explained by the transient inhibition of pSTAT1 caused by the rapid kinetic of JAK inhibition observed in *ex vitro* experiments (9). In addition, the evaluation of 6 gene-based assays is not enough because of the very small number of genes to fully reflect the activation of the IFN pathway, where hundreds of ISGs are induced, especially in different subtypes of IFNopathies, and also, no consensus exists on the groups of ISGs. In addition to the incomplete inhibition of the IFN pathway, changes in other cytokines and pathways, including IL-6, IL-1B, and NF-κB, were already observed in IFNopathies; thus, they might also lead to such a result (5, 19, 25).

Considering that jakinibs not only inhibit the IFN pathway but also have effects on other inflammatory signaling pathways such as IL-6 and IFN-γ, extra attention should be paid on the occurrence of side effects when using jakinibs (26). Several side effects have been documented in previous studies, with infections being the most remarkable. Upper respiratory tract infections are the most recorded. Sanchez et al. also observed polyomavirus viremia in patients during baricitinib treatment (15). Therefore, a regular screening of this virus should be considered, especially for SAVI patients whose underlying lung defect make them vulnerable to infections. Fortunately, the treatment with tofacitinib or ruxolitinib was overall well tolerated by our patients and no one developed severe infections during the treatment. Patient 3 who had a *cytomegalovirus* infection prior to ruxolitinib treatment did not further worsen after the start of the treatment. However, 3 patients in our study developed lymphopenia during the treatment, which was not reported in previous studies. Indeed, Sanchez et al. showed that the absolute lymphocyte count increased along with disease improvement after the treatment in patients with lymphopenia at baseline (15). In addition, considering the widespread effects of other cytokines, a cytokine rebound effect should not be ignored when the treatment is discontinued, as it was reported in a previous study (9).

Given the widespread influence on other cytokines, more selective inhibitors or other avenues are needed to avoid severe side effects. For instance, filgotinib and upadacitinib, both selective inhibitors of JAK1, are promising candidates in the future, as well as the monoclonal antibody to type I IFN receptor subunit 1 (IFNAR1) anifrolumab, which showed promising effects in systemic lupus erythematosus (27). Treatment with reverse transcriptase inhibitors also showed encouraging results in patients with AGS1-AGS5 in which the diseases are predominantly mediated *via* an RNA signaling pathway (28). Hematopoietic stem cell transplantation, widely used as a therapy able to cure other inborn errors of immunity has not been well studied in IFNopathies to date. Kataoka et al. recently reported a patient with PSMB9 mutation who was cured by hematopoietic stem cell transplantation (29). However, more studies are needed to confirm its efficacy in other IFNopathies, especially when severe neurological lesions or lung defects occur.

In conclusion, our results indicated that jakinibs including ruxolitinib and tofacitinib are promising therapies in patients with SAVI, AGS, and SPENCD, especially for cutaneous lesions and febrile attacks. Our results also demonstrated that the combined therapy is essential for these patients. Considering the heterogeneous improvement observed in previous studies, prospective studies are necessary, as well as an urgent need to explore other effective approaches.

## Data Availability Statement

The raw data supporting the conclusions of this article will be made available by the authors, without undue reservation.

## Ethics Statement

The studies involving human participants were reviewed and approved by the ethics committee of Peking Union Medical College Hospital. Written informed consent to participate in this study was provided by the participants’ legal guardian/next of kin. Written informed consent was obtained from the minor(s)’ legal guardian/next of kin for the publication of any potentially identifiable images or data included in this article.

## Author Contributions

HS designed this study, supervised the study, and revised the manuscript. WL collected clinical data, followed the patients, performed experiments, and drafted the manuscript. WW (2nd author) contributed to the genetic analysis and supervised the study. WW (3rd author), LZ, LG, JM, CW, MQ, XT, YZ, SJ, LW, and MM followed the patients. All authors have read and approved the manuscript and agree to be accountable for all aspects of the work.

## Funding

This work was supported partly by the National Key R&D Program of China (Grant number 2021YFC2702000) and the CAMS Innovation Fund for Medical Sciences (CIFMS) (Grant number 2021-I2M-C&T-B-008).

## Conflict of Interest

The authors declare that the research was conducted in the absence of any commercial or financial relationships that could be construed as a potential conflict of interest.

## Publisher’s Note

All claims expressed in this article are solely those of the authors and do not necessarily represent those of their affiliated organizations, or those of the publisher, the editors and the reviewers. Any product that may be evaluated in this article, or claim that may be made by its manufacturer, is not guaranteed or endorsed by the publisher.
