# Supplementary material for: Janus Kinase Inhibitors in the Treatment of Type I Interferonopathies: A Case Series From a Single Center in China
Source: Front Immunol. 2022 Mar 28;13:825367. doi: 10.3389/fimmu.2022.825367 (PMC8995420; doi:10.3389/fimmu.2022.825367)
Supplement: Supplementary file 1 [file DataSheet_1.docx]

***Supplementary Material***

**
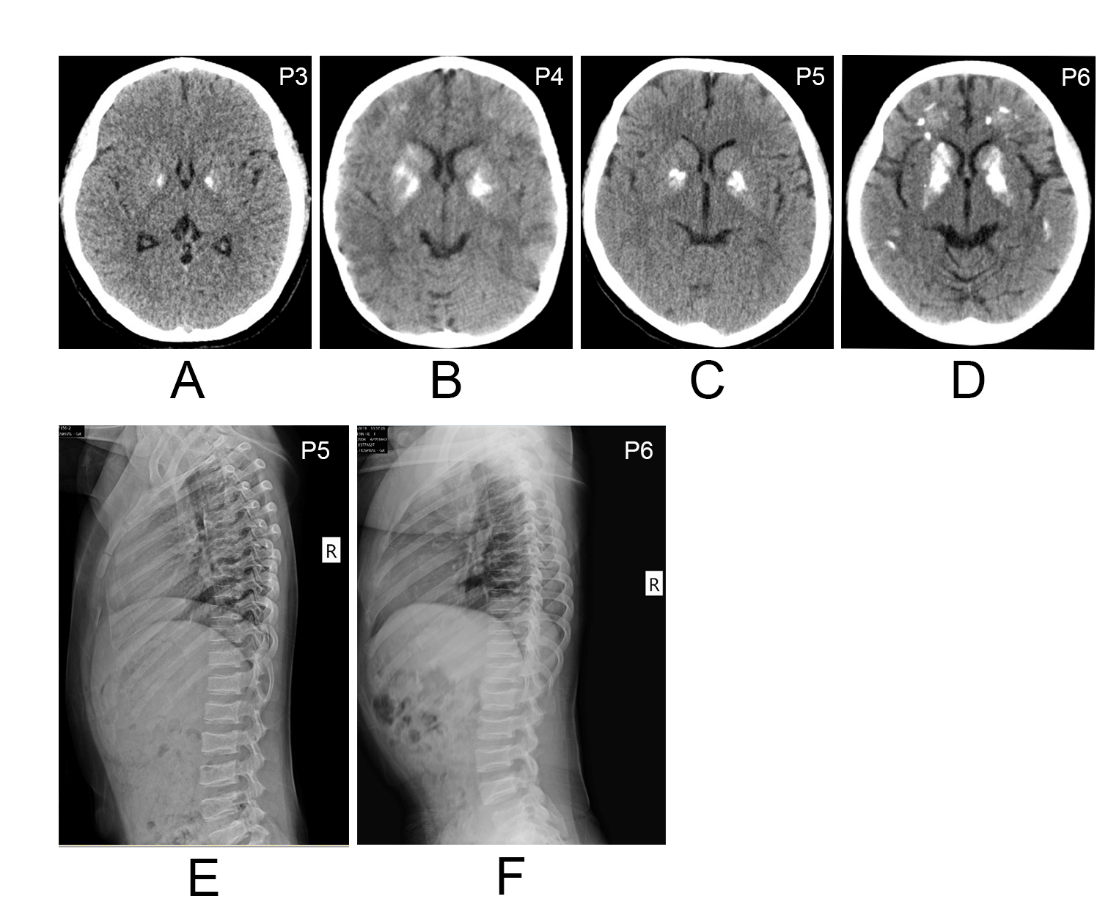
**

**Supplementary Figure 1** Calcifications in CT scan and X-ray of patients with AGS and SPENCD. Brain CT scan showing symmetric calcifications in the basal ganglia in patient 3 (**A**), patient 4 (**B**), patient 5 (**C**), and patient 6 (**D**). Plain X-ray demonstrated both platyspondyly and metaphyseal dysplasia in patient 5 (**E**) and patient 6 (**F**). P, patient.

**
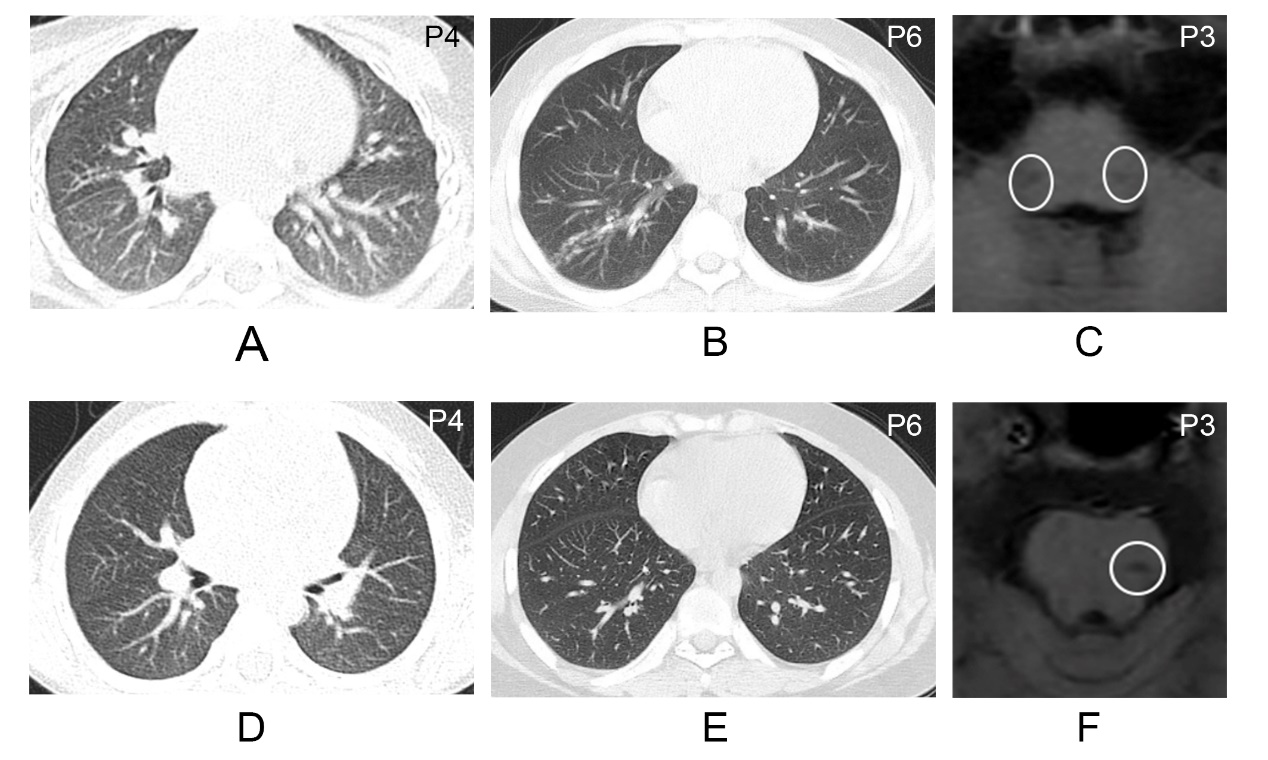
**

**Supplementary Figure 2** Chest CT scan and MRI of IFNopaties patients before and after the treatment. Chest CT scan of patient 4 (**A**) and patient 6 (**B**) demonstrating interstitial lung lesions before the treatment and no significant improvement after the treatment (**D**, **E**). Hypointense signal in bilateral cerebral peduncle in T1-weighted MRI (**C**), and disappearance of hypointense signal in right cerebral peduncle in T1-weighted MRI after the treatment in patient 3 (**F**). P, patient.

**Supplementary** **Table 1** Primers of 6 ISGs and 2 housekeeping genes for qPCR.

| Primer | Gene | Sequence |
| --- | --- | --- |
| OAZ F | OAZ | GGATAAACCCAGCGCCAC |
| OAZ R |  | TACAGCAGTGGAGGGAGACC |
| β-actin F | β-Actin | CCAACCGCGAGAAGATGA |
| β-actin R |  | CCAGAGGCGTACAGGGATAG |
| IFIT1 F | IFIT1 | AGAAGCAGGCAATCACAGAAAA |
| IFIT1 R |  | CTGAAACCGACCATAGTGGAAAT |
| IFI27 F | IFI27 | TGCTCTCACCTCATCAGCAGT |
| IFI27 R |  | CACAACTCCTCCAATCACAACT |
| IFI44L F | IFI44L | TTGTGTGACACTATGGGGCTA |
| IFI44L R |  | GAATGCTCAGGTGTAATTGGTTT |
| ISG15 F | ISG15 | GAGGCAGCGAACTCATCTTT |
| ISG15 R |  | AGCATCTTCACCGTCAGGTC |
| SIGLEC1 F | SIGLEC1 | AGCTGAGGCCAACTCCCTGA |
| SIGLEC1 R |  | AGGCTCCTCGGACCTGGAAG |
| RSAD2 F | RSAD2 | TGCTTTTGCTTAAGGAAGCTG |
| RSAD2 R |  | AGGTATTCTCCCCGGTCTTG |
